# Human Erythroid Progenitors Are Directly Infected by SARS-CoV-2: Implications for Emerging Erythropoiesis in Severe COVID-19 Patients

**DOI:** 10.1016/j.stemcr.2021.02.001

**Published:** 2021-02-05

**Authors:** Hector Huerga Encabo, William Grey, Manuel Garcia-Albornoz, Henry Wood, Rachel Ulferts, Iker Valle Aramburu, Austin G. Kulasekararaj, Ghulam Mufti, Venizelos Papayannopoulos, Rupert Beale, Dominique Bonnet

**Affiliations:** 1 Haematopoietic Stem Cell Laboratory, The Francis Crick Institute, 1 Midland Road, London NW1 1AT, UK; 2 Department of Haematology, King's College Hospital, London SE5 9RS, UK; 3 Cell Biology of Infection Laboratory, The Francis Crick Institute, London NW1 1AT, UK; 4 Antimicrobial Defense Laboratory, The Francis Crick Institute, London NW1 1AT, UK

**Keywords:** SARS-CoV-2, human hematopoietic stem/progenitor cells, erythropoiesis, anemia, hypoxia

## Abstract

We document here that intensive care COVID-19 patients suffer a profound decline in hemoglobin levels but show an increase of circulating nucleated red cells, suggesting that SARS-CoV-2 infection either directly or indirectly induces stress erythropoiesis. We show that ACE2 expression peaks during erythropoiesis and renders erythroid progenitors vulnerable to infection by SARS-CoV-2. Early erythroid progenitors, defined as CD34^−^CD117^+^CD71^+^CD235a^−^, show the highest levels of ACE2 and constitute the primary target cell to be infected during erythropoiesis. SARS-CoV-2 causes the expansion of colony formation by erythroid progenitors and can be detected in these cells after 2 weeks of the initial infection. Our findings constitute the first report of SARS-CoV-2 infectivity in erythroid progenitor cells and can contribute to understanding both the clinical symptoms of severe COVID-19 patients and how the virus can spread through the circulation to produce local inflammation in tissues, including the bone marrow.

## Highlights

• COVID19 severe patients show severe anemia and increase in nucleated red blood cells
• Erythroid progenitors co-express ACE2 and TMPRSS2
• Early and mid-late erythroid progenitors could be infected by SARS-CoV-2
• SARS-CoV-2 can be detected in erythroid colonies 14 days after the initial infection

## Graphical Abstract

**Figure:**
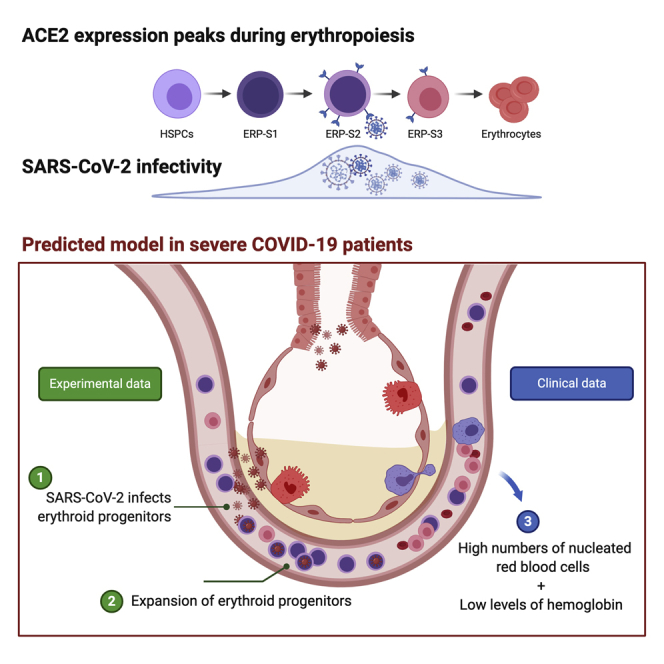

## Teaser

H. Huerga Encabo and colleagues demonstrate that SARS-CoV-2 can infect erythroid progenitors without impairing their functionality, rather causing an expansion of colonies where the virus can be detected. These findings shed light into the patients with severe COVID-19 who suffer from an aberrant increase of nucleated red blood cells concomitant with reduced levels of hemoglobin.

## Introduction

The severe acute respiratory syndrome coronavirus 2 (SARS-CoV-2) has infected and spread globally among humans, causing a pandemic crisis. The resulting Coronavirus Disease 2019 (COVID-19) is a spectrum of responses to SARS-CoV-2 infection, from asymptomatic individuals to severe disease and mortality (Guan et al., 2020).

The mechanism of infection by SARS-CoV-2 is fairly well characterized. For viral entry, the binding between the viral surface spike glycoprotein (S) and target cell surface angiotensin-converting enzyme-2 (ACE2) followed by cleavage of S by the transmembrane protease serine 2 (TMPRSS2) are required (Walls et al., 2020). ACE2 expression has been extensively reported in different epithelial cells from the respiratory tract, constituting the main infection site of SARS-CoV-2 (Lukassen et al., 2020).

Further reports demonstrate ACE2 expression in cells from other tissues such as intestinal epithelial cells, hepatocytes, and neurons, making these cells vulnerable to be infected by SARS-CoV-2 (Pellegrini et al., 2020; Sungnak et al., 2020; Wang et al., 2020; Zhang et al., 2020). In line with these findings, increasing evidence of gastrointestinal, hepatic, and neurological symptoms have been reported (Varatharaj et al., 2020; Wu et al., 2020; Yang and Tu, 2020). More recently, an aberrant increase of erythroid progenitors in circulation has been reported, especially in severe cases (Bernardes et al., 2020). Indeed, this observation together with hypoxia, anemia, and coagulopathies highly correlate with severity and mortality (Bernardes et al., 2020; Vabret et al., 2020). Disruption of erythropoiesis may be an indirect effect of the systemic hyperinflammation that occurs in intensive care patients. Another potential explanation is that direct targeting of hematopoietic stem/progenitor cells (HSPCs) and/or erythroid progenitors (ERP) by SARS-CoV-2 contributes to the hematological features of COVID-19. It has previously been suggested that SARS-CoV-2 can have a tropism for blood cells (Cavezzi et al., 2020). More recently, HSCs from cord blood have been reported to express ACE2, and exposure to the Spike protein can reduce their functionality (Ropa et al., 2020). Despite these observations, the infectivity of SARS-CoV-2 in HSPCs and more particularly in erythroid progenitors has not been characterized. In this report, we have studied HSPCs and different erythroid progenitor populations to assess if they can be infected by SARS-CoV-2.

## Results

**Figure 1:**
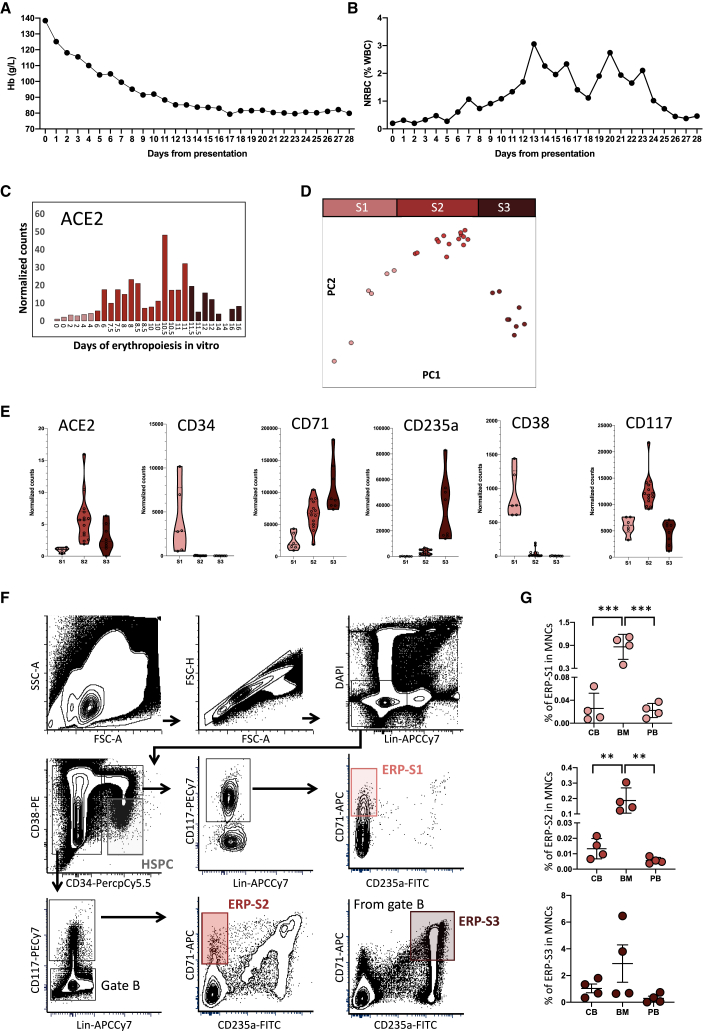
COVID-19 Patients Show Symptoms of Disruptive Erythropoiesis and ACE2 Is Highly Expressed in Erythroid Progenitors (A and B) Monitoring of the hemoglobin levels (A) and nucleated red blood cells (B) during the first 28 days post hospitalization at King's College Hospital. Data represent the mean value of the 30 patients for each day. (C) ACE2 expression during *in vitro* erythropoiesis (from GSE118537). Each bar represents the normalized counts for ACE2 of each individual sample during the time course. (D) Principal component analysis of GSE118537 dataset with the three subpopulations analyzed. Time points of S1 are represented in pink, time points of S2 in red, and time points of S3 in garnet. (E) Expression level of ACE2 and classical markers to define HSPCs and erythroid progenitors in S1, S2, and S3. Each dot represents one sample of the time course. (F) Representative gating strategy from bone marrow to define ERP-S1, ERP-S2, and ERP-S3. Lineage cocktail includes CD14, CD16, and CD19. (G) Percentage of each erythroid progenitor in the mononuclear cell fraction of each tissue analyzed (CB, cord blood; BM, bone marrow; PB, peripheral blood). Each dot represents one independent human donor. Two-way ANOVA test was used for the comparison among the three tissues; ^∗∗^p < 0.01; ^∗∗∗^p < 0.005.

Patients treated for severe COVID-19 (as defined by requirement for intensive care admission) at King's College Hospital (London, UK) show a rapid and profound decline in hemoglobin following admission (Figure 1A). Interestingly, this is associated with the appearance of circulating nucleated red cells, peaking between 2 and 3 weeks after admission (Figure 1B). Numbers of white cells and platelets rise over this period, indicating that the anemia is not associated with myelosuppression (Figures S1A and S1B).

Publicly available datasets indicate that ACE2 and TMPRSS2 expression is scarce among hematopoietic cell types, and absent on human HSPCs. Of note, progenitors of the erythroid lineage appear to be the only cell types expressing both ACE2 and TMPRSS2 among the cells present in the bone marrow (The Human Cell Atlas Bone Marrow Single-Cell Interactive Web Portal). Intrigued by this observation, we used detailed datasets of *in vitro* human erythropoiesis (Gillespie et al., 2020) to characterize ACE2 expression from HSPCs, CD34^+^ cells, to mature erythrocytes. We found that ACE2 expression peaks during erythropoiesis (Figure 1C). During the first days of erythropoiesis ACE2 is not expressed, starting to be expressed around day 6 to 7, before reaching its maximum level of expression at day 10 to 11 and then declining until the last time point at day 16. Therefore, we can distinguish three stages of ACE2 expression, where Stage 1 (S1) represents the initial time points with no ACE2 expression, Stage 2 (S2) represents the time points where ACE2 expression increases and reaches maximum levels, and Stage 3 (S3) represents the last time points where ACE2 expression is declining and at low levels. Accordingly, in our analysis of the differentially expressed genes among these three stages, ACE2 appeared as one of the top upregulated genes in S2 (Figure S1C). Interestingly, these three stages coincide with three clusters observed by the dimensionality reduction analysis during the erythroid differentiation (Figure 1D). S1 differentially expressed genes include stem and early progenitor markers such as CD34, CD38, and RUNX1. In S2, these markers disappear and erythroid-committed progenitor markers like CD71 and MYB are upregulated. Finally, in S3, GATA1 and KLF1 target genes are enriched (Figures S1C and S1D, Table S1) as expected in late and terminal erythroid differentiation (Ludwig et al., 2014).

Based on the expression of different surface markers frequently used in flow cytometry to isolate different erythroid progenitors (Chen et al., 2009; Mello et al., 2019; Westers et al., 2017), we defined three erythroid progenitor populations (ERP) in order to isolate S1 to S3 from different tissues (Figure 1E). Erythroid progenitors of S1 (ERP-S1) can be defined as CD34^+^CD117^+^CD71^+^CD235a^−^, ERP-S2 as CD34^−^CD117^+^CD71^+^CD235a^−^ and ERP-S3 as CD34^−^CD117^−^CD71^+^CD235a^+^ (Figure 1F). Of note, using another independent dataset, we also observed that ACE2 expression is higher in erythroid progenitors when defined as CD71^+^CD235a^−^, further validating our strategy to enrich for ACE2^+^ erythroid progenitors (Ludwig et al., 2019).

We characterized and measured the abundance of ERP-S1, ERP-S2, and ERP-S3 in cord blood, bone marrow, and peripheral blood (Figure 1G). As expected, all types of erythroid progenitors are more abundant in the bone marrow. Nonetheless, due to the possible impact as a SARS-CoV-2 chaperone, is worth highlighting that ACE2^+^ erythroid progenitors are also present in peripheral blood (Figure S1E).

**Figure 2:**
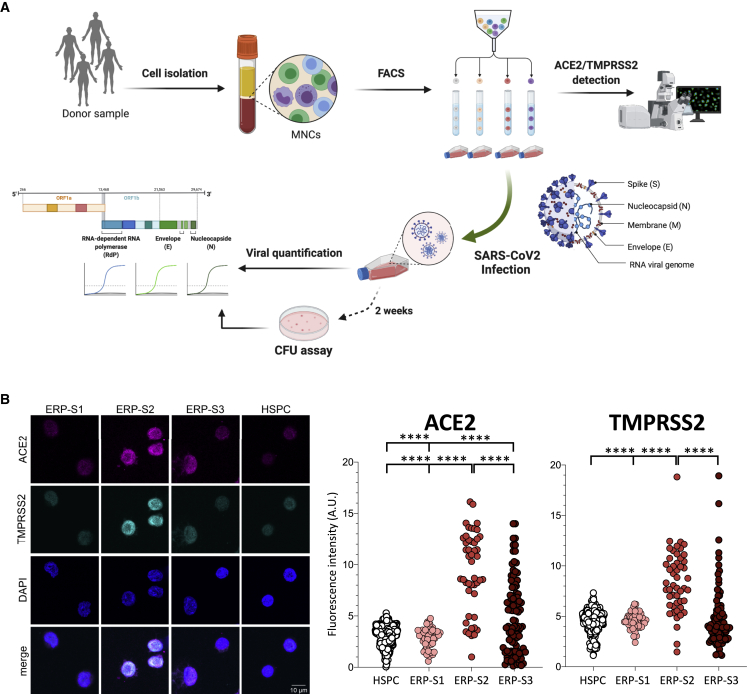
Isolation and Analysis of Erythroid Progenitors co-expressing ACE2 and TMPRSS2 (A) Schematic of sample processing: human bone marrow or peripheral blood were processed to isolate by cell sorting ERP populations. ACE2 and TMPRSS2 at the cell membrane was analyzed by confocal microscopy. SARS-CoV-2 infectivity was quantified by the detection of three viral genes (N, Nucleocapsid; E, Envelope; and RdRP, RNA-dependent Polymerase) by real-time qPCR. Created with BioRender. (B) Immunostaining and fluorescence intensity quantification of ACE2 and TMPRSS2 in HSPCs and erythroid progenitors (ERPs) (scale bar, 10 μm). Each dot represents one cell; HSPCs (351 cells), ERP-S1 (50 cells), ERP-S2 (48 cells), ERP-S3 (106 cells). This is representative of one of the two independent immunostainings performed. Two-way ANOVA test was used for the comparison among the different cell populations; ^∗∗∗∗^p < 0.001.

From bone marrow aspirates of human healthy donors, we used our markers to isolate the three ERP populations by cell sorting, determine the expression of ACE2 and TMPRSS2, and evaluate their susceptibility to infection by SARS-CoV-2 (Figure 2A). As expected, when we analyzed the expression of ACE2 at the RNA level, the ERP-S2 population showed the highest level of expression while ERP-S3 showed low levels and both ERP-S1 and HSPCs showed no expression of ACE2 (Figure S2A). More importantly at the protein level, the immunofluorescence analysis of the different ERP populations confirmed that ACE2 and TMPRSS2 are co-expressed in the ERP-S2 cells (Figures 2B, S2B, and S2C). Notably, we also detected a subset of ERP-S3 cells that co-expressed ACE2 and TMPRSS2, while we did not detect any cell in the ERP-S1 and HSPC populations showing ACE2/TMPRSS2 co-expression (Figures 2B, S2B, and S2C).

**Figure 3:**
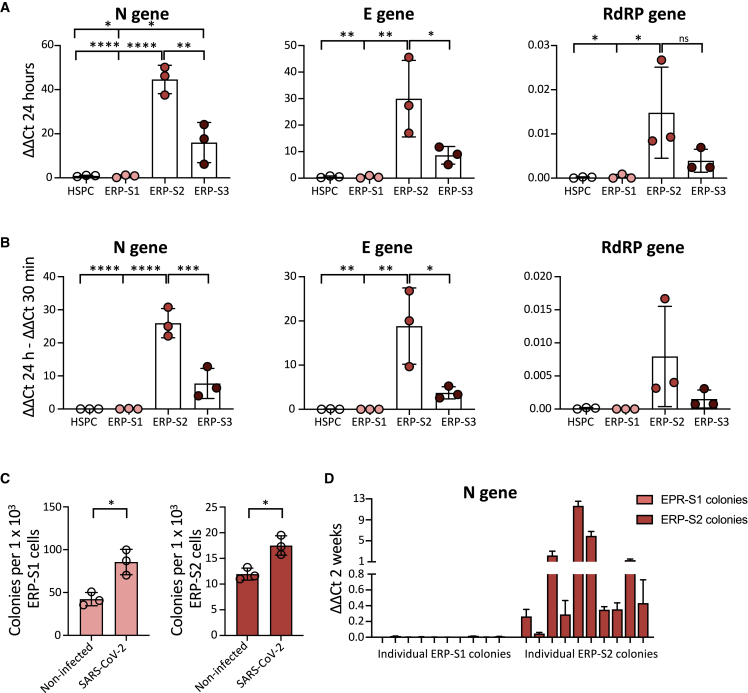
SARS-CoV-2 Infects Erythroid Progenitors and can be Detected in Erythroid Colonies 14 Days Later (A) SARS-CoV-2 infection at MOI 5 in ERPs from bone marrow. Quantification by real-time qPCR of the Nucleocapsid (N), Envelope (E), and RNA-dependent RNA Polymerase (RdRP) SARS-CoV-2 genes at 24 h post infection. Each dot represents an independent biological donor (n = 3). Values represent ΔΔCt normalized to GAPDH. Error bars show the mean ± SD. Two-way ANOVA test was used for the comparison among the different cell populations; ^∗^p < 0.05; ^∗∗^p < 0.01; ^∗∗∗^p < 0.005; ^∗∗∗∗^p < 0.001; ns, no significance. (B) Replication of SARS-CoV-2 genes between 30 min and 24 h post infection. Each dot represents an independent biological donor (n = 3). Values represent the differences in ΔΔCt normalized to GAPDH between the quantification of viral genes at 24 h and quantification after 30 min of viral infection. Error bars show the mean ± SD. Two-way ANOVA test was used for the comparison among the different cell populations; ^∗^p < 0.05; ^∗∗^p < 0.01; ^∗∗∗^p < 0.005; ^∗∗∗∗^p < 0.001. (C) CFU assay of ERPs after 24 h in culture with the virus. Data represent total number of colonies per 1000 ERP-S1 cells (left) or ERP-S2 cells (right); 4,000 cells of each population were seeded in triplicates in methylcellulose plates and colonies were counted 14 days later. Each dot represents an independent biological donor (n = 3). Error bars show the mean ± SD. The t test was used for the comparison between the different conditions of each sample; ^∗^p < 0.05. (D) SARS-CoV-2 detection (N gene) in 10 independent colonies from ERP-S1 or ERP-S2 plates. After 14 days we pick colonies, extract RNA, and analyze by real-time qPCR the presence of the virus. Data represent the mean ± SD of the real-time qPCR triplicates for each independent colony of one experiment.

We then performed infection assays on bone marrow HSPCs and the three ERP populations. To analyze the capacity of the virus to directly infect these cells, we measured different viral genes by real-time qPCR in the different populations after being in the presence of SARS-CoV-2. Interestingly, we observed that ERP-S2 cells are highly susceptible to SARS-CoV-2 infection, as shown by the detection of different viral genes after 24 h of infection (Figure 3A). In contrast, HSPCs and ERP-S1 cells are completely refractory to infection by SARS-CoV-2, as we cannot detect any trace of the virus in these cells. In keeping with the ACE2/TMPRSS2 cell surface levels, we also detected viral genome in ERP-S3 cells, although at a lower level, indicating that erythroid progenitors in this stage of differentiation are also susceptible to infection. To address the question of whether SARS-CoV-2 is not only able to bind to ERP-S2 cells (and to a lesser extent to ERP-S3 cells), but is also capable of replicating inside these progenitor cells, we analyzed the difference in the viral genome load between 30 min (where we just detect viruses putatively attached to cells) and 24 h (where we also quantify viruses amplified inside cells) after exposure. Importantly, our results showed that SARS-CoV-2 can infect and amplify its genome in erythroid progenitors of S2 and S3 but is not able to bind and infect HSPCs from the bone marrow (Figure 3B). To reproduce more physiological scenarios, we also infected the erythroid progenitors from peripheral blood from healthy individuals at lower multiplicity of infection (MOI) and we obtained similar results (Figure S3A). We next performed colony-forming unit (CFU) assay to analyze how the exposure to SARS-CoV-2 could influence the functionality of these erythroid progenitor populations. As expected, ERP-S1 and ERP-S2 are able to generate colonies, unlike the more differentiated ERP-S3 population, and ERP-S1 is more prompted to generate burst-forming unit (BFU)-E colonies while ERP-S2 is more prompted to generate CFU-E colonies (Figure S3B). After being in the presence of SARS-CoV-2, both erythroid progenitor populations produce more colonies (Figure 3C). We were intrigued by the possibility that after the 2 weeks of CFU experiment the virus could remain in these cells/colonies. To address this question, we picked independent colonies from ERP-S1 or ERP-S2 plates and analyzed the presence of SARS-Cov-2 virus by real-time qPCR. While we were not able to amplify viral genes in any colony from ERP-S1 (0 out of 10 in two independent experiments), we detected the virus in ∼90% of the colonies from ERP-S2 (Figures 3D and S3C) (9 of 10 in experiment 1 and 8 of 10 in experiment 2 [data not shown]). Interestingly, these results indicate that SARS-CoV-2 remains in ERP-S2 after 14 days of the initial infection.

## Discussion

The COVID-19 pandemic continues to constitute a huge threat to public health worldwide. Despite the efforts and advances to untangle the mechanisms of SARS-CoV-2 infection and transmission among humans, we are still blind to the overall COVID-19 pathology and its consequences. The results we present here might help understand the emergent erythropoiesis and aberrant presence of erythroid progenitors in the peripheral blood of patients with severe COVID-19 (Bernardes et al., 2020; Shahbaz et al., 2020). This recent evidence indicates that the increase of erythroid progenitors in circulation constitute a hallmark of both severity and fatality in patients with COVID-19 (Bernardes et al., 2020; Shahbaz et al., 2020). In this context, our observations that the virus can be detected after 14 days in the ERP-S2 without impairing their viability, rather causing an increase of colonies, suggest that the expansion of circulating erythroid cells that have been reported in severe patients may be due to direct infection of upstream CD71^+^CD235a^−^ progenitors. In-depth research will be key to elucidate which of these two events, direct infection of erythroid progenitors and pathological increase of these populations in the bloodstream, occur first *in vivo* in patients. Our results also open the question about the causality between direct SARS-CoV-2 infection of erythroid progenitors and clinical manifestations resulting from hemoglobin decline and anemia. In light of our findings, we cannot discard the possibility that these symptoms are not simply a byproduct of inflammation and overall poor health and that, instead, direct infection of erythroid progenitors by SARS-CoV-2 contributes to this aberrant erythropoiesis.

The results we show here constitute the first evidence of direct infection of specific erythroid progenitors, named as ERP-S2 (CD71^+^CD235A^−^) and ERP-S3 (CD71^+^CD235a^+^), by SARS-CoV-2. Especially relevant is the high ability (or facility) of the virus to infect ERP-S2, the most vulnerable erythroid progenitor population. Based on previous works and our observations, ERP-S2 population includes CFU-E cells and early pro-erythroblast cells (Ludwig et al., 2019; Westers et al., 2017). Considering their high proliferative capacity, the infection of these progenitors by SARS-CoV-2 may have a major detrimental impact not only in erythropoiesis, but also in the spread of the virus through millions of circulating infected cells. Also, this presents a scenario in which infected erythroid progenitors may cause local inflammation in the bone marrow, which could cause a drastic disruption of hematopoiesis and the production of immune cells.

In contrast to the observations in erythroid progenitors, we report that bone marrow HSPCs (CD34^+^CD38^−^) do not express ACE2 and TMPRSS2 at the RNA or protein levels. Consequently, we show that HSPCs are not infected by SARS-CoV-2.

In this report, we also provide clinical data of a cohort of 30 patients with COVID-19 who were treated in intensive care units (ICU) at King's College London. We show that the decline in hemoglobin levels coincides with an aberrant increase of nucleated red blood cells in circulation. These nucleated red blood cells correspond to ERP-S3 cells (Shahbaz et al., 2020). Similar to what we show here, Shahbaz and colleagues also report that this population can be infected by SARS-CoV-2 and that the infection induces the immunosuppressive capacity of these cells. Of note, they did not analyze the population that we characterize here as ERP-S2, the more upstream progenitor population, which is more susceptible to infection (CD71^+^CD235a^−^). Importantly, our results might also have an impact in our understanding of the transmission and incubation period of SARS-CoV-2, as different levels of infected progenitors in the bloodstream could greatly alter such parameters. Finally, identification of infected ERP-S2 populations in hospitalized patients could help identify those patients who will suffer from severe hematopathology, potentially allowing preemptive management strategies to improve outcomes.

## Experimental Procedures

### Clinical Data

A sample of 30 patients who were being treated for COVID-19 in ICUs at King's College Hospital on May 1, 2020, and who had first tested positive for SARS-CoV-2 by PCR within 7 days of admission to hospital, was identified using information from the hospital's Business Intelligence Unit and electronic patient record (EPR) system. Values for peripheral blood hemoglobin concentration, white blood cell count, platelet count, and nucleated red blood cell percentage on the day of presentation to hospital and subsequent 28 days were collected from the EPR system. In cases where multiple tests were performed in the same day, the first value was collected.

### RNA Sequencing Data Import and Analysis of ACE2 Expression

RNA sequencing dataset was downloaded from GEO database (GEO accession number GSE118537).

Differential gene expression analysis between erythroid subpopulations was performed using DEseq2 methodology on a pairwise comparison fashion. All genes with an adjusted p value <0.05 were considered as statistically significant between two subpopulations. Each of the three ERP populations was compared with the other two and those genes differentially expressed in both comparisons were considered as differentially expressed for the specific subpopulation. Top 300 upregulated genes in each ERP population are listed in Table S1.

### Isolation of Mononuclear Cells from Human Cord Blood, Bone Marrow, and Peripheral Blood

Umbilical cord blood was obtained from full-term donors after informed consent at the Royal London Hospital (London, UK). The collection and use of these samples was approved by the East London Research Ethical Committee (REC:06/Q0604/110) and in accordance with the Declaration of Helsinki. Mononuclear cells were isolated by density centrifugation using Ficoll-Paque (GE 67 Healthcare). Human bone marrow mononuclear cells were obtained from StemCell Technologies (Cat# 70001). Peripheral blood was isolated from consenting unscreened healthy adult volunteers following approved protocols by the ethics board of the Francis Crick Institute and the regulations of the Human Tissue act 2004. Peripheral blood mononuclear cells were isolated by centrifugation over a Histopaque-1119 gradient (Sigma-Aldrich 11,191).

### mRNA Quantification by Real-time qPCR

Total RNA was extracted using the RNeasy Microkit (Qiagen, Cat# 74,004), and the RNA was retrotranscribed using the SuperScript III First-Strand Synthesis system (Thermo Fisher Scientific, Cat# 18080051). For real-time qPCR, PowerUP SYBR Green (Applied Biosystems, Cat# 15310939), MicroAmp Optical 384-Well Reaction Plate (Applied biosystems, Cat# 4309849), and the Applied Biosystems QuantStudio 7 were used according to the instructions provided by the manufacturers. For the detection of SARS-CoV-2 we used previously validated primers (Corman et al., 2020). See primers used in the Supplementary Information.

### Flow Cytometry Analysis and Cell Sorting

All experiments were analyzed at the Flow Cytometry core facility of The Francis Crick Institute using the LSR FORTESSA (BD Biosciences) equipped with a 488-nm laser, a 561-nm laser, a 633-nm laser, and a 405-nm laser. For sorting, cell suspensions were filtered through a 35-μm nylon mesh (Falcon, Cat# 352235) and sorted in a BD FACS FUSION cell sorter equipped with 488-nm, 561-nm, 633-nm, and 405-nm lasers. See antibodies used in the Supplementary Information. All experiments were analyzed with FACSDiva 6.2 (BD Biosciences) and FCS Express 7 software.

### Immunostaining, Confocal Microscopy, and Immunofluorescence Quantification

Polyclonal goat anti-ACE2 antibody (R&D Systems, Cat# AF933) at 15 μg/mL and polyclonal rabbit anti-TMPRSS2 (ThermoFisher, Cat# PA5-14264) at 15 μg/mL were used for the primary antibody incubation. Donkey anti-goat Alexa 647 (Invitrogen, Cat# A21447) and donkey anti-rabbit Alexa 488 (Invitrogen, Cat# A21206) were used at 10 μg/mL for the secondary antibody staining. Fluorescence imaging was performed at a Leica TCS SP5 inverted confocal microscope using the sequential scan in between frames mode with a ×63 objective. Background signal was accounted for by performing secondary only staining controls. Fiji/ImageJ version 2.0.0 software was used for image analysis. Fluorescence intensity quantifications were done by selecting individual cells using an intensity-based mask at the DAPI channel of at least five different representative regions of interest per group of cells. This mask was then applied for the quantification of fluorescence intensity in the 488 channel (TMPRSS2) and the 647 channel (ACE2).

### SARS-CoV-2 Production and Infection

Vero E6 cells (a kind gift from Oliver Schwarz, Institute Pasteur, Paris) were maintained in DMEM modified with high glucose, L-glutamine, phenol red and sodium pyruvate (ThermoFisher, Cat# 41966-029) supplemented with 10% fetal calf serum (FCS). SARS-CoV-2 strain BetaCoV/England/02/2020 (obtained from Public Health England) was propagated at 37°C on VeroE6 cells in DMEM supplemented with 2% FCS. The titer was determined by plaque assay as follows: confluent monolayers of VeroE6 cells grown on six-well plates were incubated with 200 μL of a 10-fold serial dilution of virus stock in DMEM supplemented with 10% FCS for 1 h at room temperature. The cells were then overlaid with 0.5× DMEM supplemented with 1% FCS and 1.2% Avicel (BMC Biopolymers, Belgium). After 4-d incubation at 37°C, cells were fixed with 4% formaldehyde in PBS followed by staining with 0.1% toluidine blue (Sigma, Cat# 89640). The titer was calculated as plaque-forming units (PFU) per mL. Hematopoietic cells were infected with SARS-CoV-2 at a MOI of 5 PFU per cell. Cells were washed three times with PBS to remove unbound virus prior to lysis for RNA isolation.

### CFU assay

After 24 h of infection, cells were seeded for 2 weeks in methylcellulose-based medium with recombinant cytokines for human cells (StemCell Technologies, Cat# 04435). Two independent experiments with two to three biological donors in each experiment were analyzed to assess the number of CFUs in each cell population.

### Statistical Analysis

Statistical methods relevant to each figure are outlined in the figure legend. Sample size was not predetermined. Data are presented as means with SD to indicate the variation within each experiment. A two-way ANOVA test was used for the comparison between the different cell populations.

## Author Contributions

Conceptualization: H.H.E., W.G., and D.B.; Methodology: H.H.E., W.G., R.U., and I.V.A.; Analysis and interpretation: H.H.E., W.G., M.G.A., H.W., R.U., I.V.A., and D.B.; Patient data analysis: A.G.K., G.M., and H.W.; Writing paper: H.H.E. and D.B.; Review & Editing paper: H.H.E., W.G., M.G.A., H.W., V.P., R.B., and D.B.; Funding Acquisition: D.B.

## Supplemental Information

Document S1. Supplemental Experimental Procedures and Figures S1–S3

Table S1. Top 300 Upregulated Genes in Each ERP Population

Document S2. Article Plus Supplemental Information

## Footnotes

Supplemental Information can be found online at https://doi.org/10.1016/j.stemcr.2021.02.001.
