# Supplementary material for: Human Erythroid Progenitors Are Directly Infected by SARS-CoV-2: Implications for Emerging Erythropoiesis in Severe COVID-19 Patients
Source: Stem Cell Reports. 2021 Feb 5;16(3):428–36. doi: 10.1016/j.stemcr.2021.02.001 (PMC7862909; doi:10.1016/j.stemcr.2021.02.001)
Supplement: Document S1. Supplemental Experimental Procedures and Figures S1–S3 [file mmc1.pdf]

**Stem Cell Reports, Volume *16***

## Supplemental Information

# Human Erythroid Progenitors Are Directly Infected by SARS-CoV-2: Implications for Emerging Erythropoiesis in Severe COVID-19 Patients

**Hector Huerga Encabo, William Grey, Manuel Garcia-Albornoz, Henry Wood, Rachel Ulferts, Iker Valle Aramburu, Austin G. Kulasekararaj, Ghulam Mufti, Venizelos Papayannopoulos, Rupert Beale, and Dominique Bonnet**

# Figure S1

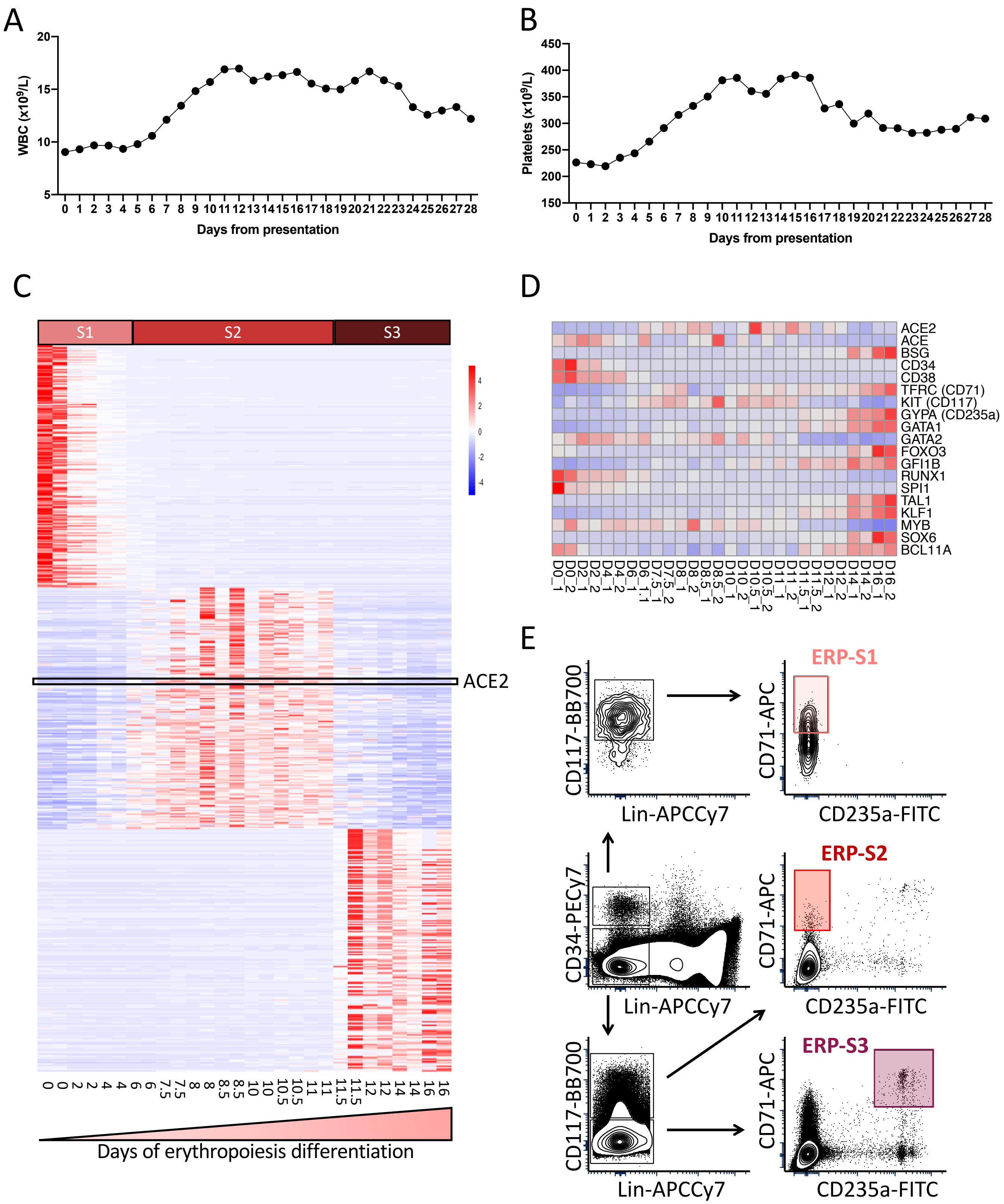
A
WBC (x10⁹/L)
Days from presentation
B
Platelets (x10⁹/L)
Days from presentation
C
S1
S2
S3
ACE2
Days of erythropoiesis differentiation
D
ACE2
ACE
BSG
CD34
CD38
TFRC (CD71)
KIT (CD117)
GYPA (CD235a)
GATA1
GATA2
FOXO3
GFI1B
RUNX1
SPI1
TAL1
KLF1
MYB
SOX6
BCL11A
E
CD117-BB700
Lin-APCCy7
CD71-APC
CD235a-FITC
ERP-S1
CD34-PECy7
ERP-S2
ERP-S3

**Figure S1. Clinical data of COVID19 patients and characterization of three main stages during the erythroid differentiation in vitro and in vivo. Related to Figure 1.**

(A-B) Monitoring of the white blood cells levels (A) and platelets (B) during the first 28 days post hospitalization at King's College Hospital. Data represents the mean value of the 30 patients for each day.

(C) Heatmap of the top 300 upregulated genes in each stage of the erythroid differentiation. ACE2 is highlighted as one of the most upregulated genes in Stage 2.

(D) Detailed heatmap of erythroid markers and transcription factors associated to early and late stages of the erythropoiesis.

(E) Gating strategy to isolate ERPs from peripheral blood of healthy individuals. Lineage cocktail includes CD14, CD16 and CD19.

# Figure S2

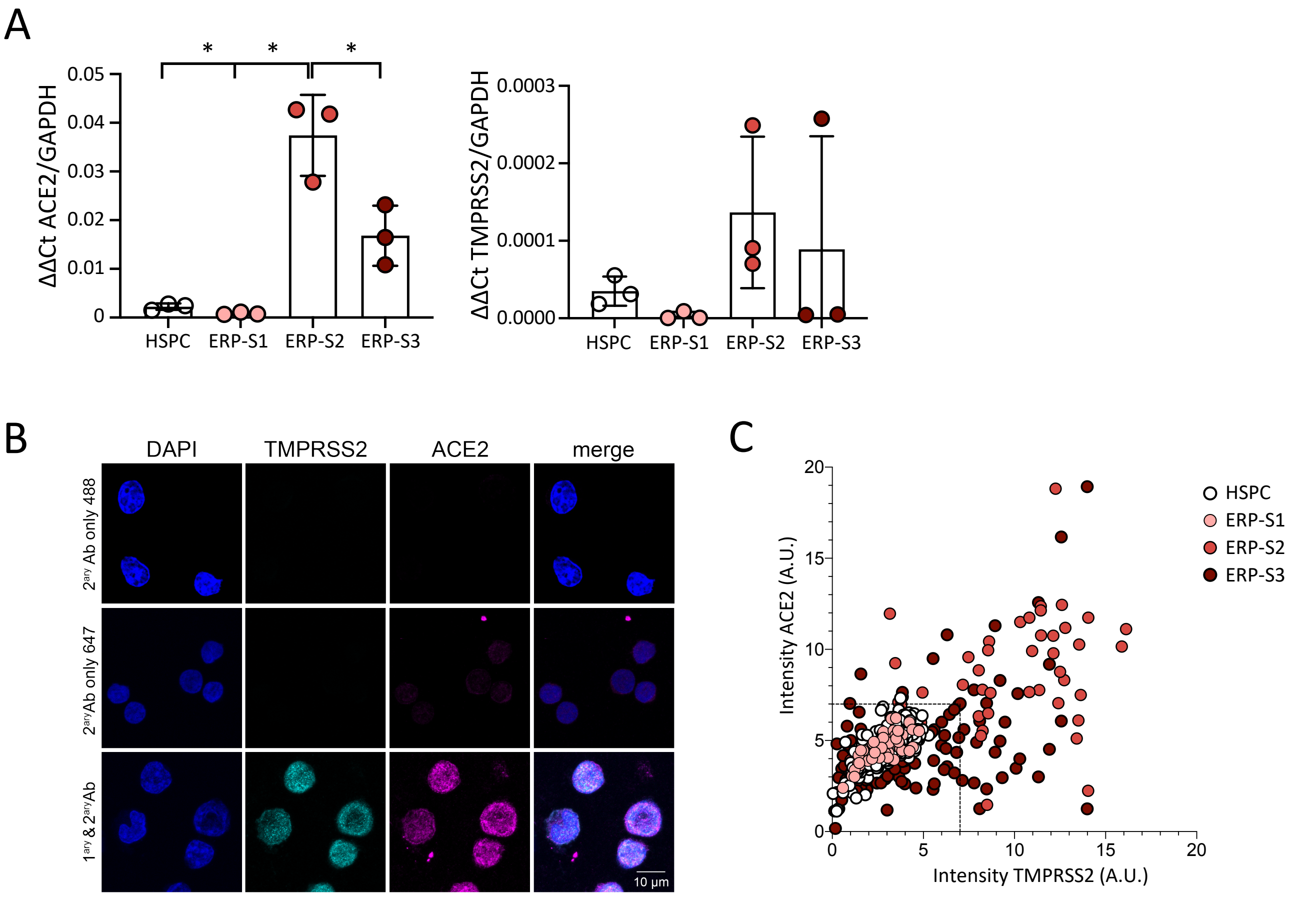
A
ΔΔCt ACE2/GAPDH
0.05
0.04
0.03
0.02
0.01
0
HSPC
ERP-S1
ERP-S2
ERP-S3
ΔΔCt TMPRSS2/GAPDH
0.0003
0.0002
0.0001
0.0000
B
DAPI
TMPRSS2
ACE2
merge
2ary Ab only 488
2aryAb only 647
1ary & 2aryAb
10 µm
C
Intensity ACE2 (A.U.)
Intensity TMPRSS2 (A.U.)
20
15
10
5
0
HSPC
ERP-S1
ERP-S2
ERP-S3

**Figure S2. Detection of ACE2 and co-expression with TMPRSS2 in erythroid progenitors. Related to Figure 2.**

(A) Quantification by RT-qPCR of ACE2 (left panel) and TMPRSS2 (right panel) in HSPCs and ERPs from human bone marrow. Each dot represents 1 independent biological replicate performed in two independent experiments. Values represent ΔΔCt normalized to GAPDH. Error bars show the mean ± SD. Two-way ANOVA test was used for the comparison among the different cell populations; $*p < 0.05$.

(B) Immunostaining controls for the detection of ACE2 and TMPRSS2 (scalebar 10 µm). Related to Figure 2B.

(C) Correlation of ACE2 and TMPRSS2 expression at protein level in HSPCs and ERPs. Related to Figure 2B. Each dot represents one cell. This is representative of one experiment of the two independent immunostainings performed.

Figure S3

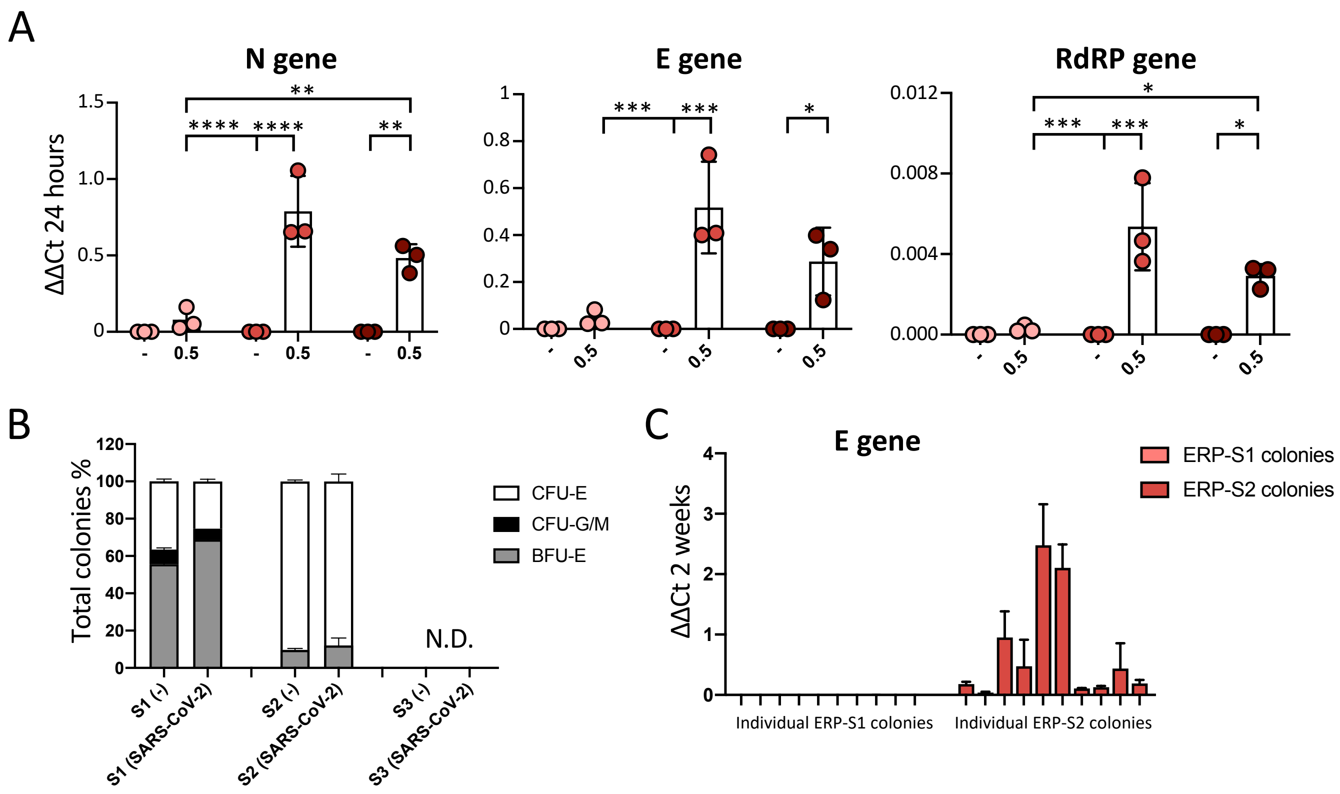
A
N gene
E gene
RdRP gene
ΔΔCt 24 hours
1.5
1.0
0.5
0
1
0.8
0.6
0.4
0.2
0.012
0.008
0.004
0.000
**
****
****
***
*
-
0.5
B
Total colonies %
CFU-E
CFU-G/M
BFU-E
N.D.
S1 (-)
S1 (SARS-CoV-2)
S2 (-)
S2 (SARS-CoV-2)
S3 (-)
S3 (SARS-CoV-2)
C
E gene
ΔΔCt 2 weeks
ERP-S1 colonies
ERP-S2 colonies
Individual ERP-S1 colonies
Individual ERP-S2 colonies

**Figure S3. Erythroid progenitors from peripheral blood capable of colony formation are susceptible to be infected at low MOI. Related to Figure 3.**

(A) SARS-CoV-2 infection at MOI 0.5 in ERPs from peripheral blood. Quantification by RT-qPCR of the Nucleocapsid (N), Envelope (E) and RNA-dependent RNA Polymerase (RdRP) SARS-CoV-2 genes at 24 hours post-infection. Each dot represents 1 independent biological donor (n=3). Values represent ΔΔCt normalized to GAPDH. Error bars show the mean ± SD. Two-way ANOVA test was used for the comparison among the different cell populations; *$p < 0.05$; **$p < 0.01$; ***$p < 0.005$; ****$p < 0.001$; ns: no significance.

(B) Percentage of types of colonies produced by each ERP population. No colonies were detected from ERP-S3 plates. 4000 cells of each population were seeded in methylcellulose plates and colony number and phenotype were determined 14 days later. Data represents the percentage of each type of colony shown in Figure 3C. ND: non-detected.

(C) SARS-CoV-2 detection (E gene) in 10 independent colonies from ERP-S1 or ERP-S2 plates. After 14 days we pick colonies, extract RNA and analyze by RT-qPCR the presence of the virus. Data represents the mean ± SD of the RT-qPCR triplicates for each independent colony of one experiment.

# Supplemental Experimental Procedures

### RNAseq data import and analysis

Raw counts were obtained via http as txt file and then analyzed using Deseq2 package in an R environment. Normalization was performed using day of development as experimental design. Deseq2 normalized counts were obtained. Unsupervised clustering was then performed with the regularized log transformed normalized counts obtained from the rlogTransformation option from Deseq2 using Ward as agglomeration method. Principal component analysis was performed for the first two principal components using the regularized log transformed normalized counts obtained from the rlogTransformation option in Deseq2.

### Primer Sets used:

| |
|---|
| ACE2_forward: 5’-GCTGCACAACCTTTTCTGCT-3’ |
| ACE2_reverse: 5’-AAATGCTTAGGTGTGGCTGC-3’ |
| TMPRSS2_forward: 5’-GTACCTGCATCAACCCCTCT-3’ |
| TMPRSS2_reverse: 5’-TATAGCCCATGTCCCTGCAG-3’ |
| SARS-CoV2_N_forward: 5’-CACATTGGCACCCGCAATC-3’ |
| SARS-CoV2_N_reverse: 5’-GAGGAACGAGAAGAGGCTTG-3’ |
| SARS-CoV2_E_forward: 5’-ACAGGTACGTTAATAGTTAATAGCGT-3’ |
| SARS-CoV2_E_reverse: 5’-ATATTGCAGCAGTACGCACACA-3’ |
| SARS-CoV2_RdRP_forward: 5’-GTGARATGGTCATGTGTGGCGG-3’ |
| SARS-CoV2_RdRP_reverse: 5’-CARATGTTAAASACACTATTAGCATA-3’ |
| GAPDH_forward: 5’-GGAGCGAGATCCCTCCAAAAT-3’ |
| GAPDH_reverse: 5’-GGCTGTTGTCATACTTCTCATGG-3’ |

### Flow cytometry antibodies used:

| |
|---|
| CD34-PECy7 (clone 4H11, eBioscience) |
| CD34- PerCP-Cy5.5 (clone 8G12, BD Pharmingen) |
| CD38-PE (clone HIT2, BD Pharmingen) |
| CD117-BB700 (clone YB5.B8, BD Bioscience) |
| CD117-PECy7 (clone 104D2, eBioscience) |
| CD71-APC (clone OKT9, eBioscience) |
| CD235a-FITC (clone HIR2, BD Pharmingen) |
| DAPI (1 µg/ml; BD Biosciences, Cat# 564907) |
